# Author Correction: Iron restriction inside macrophages regulates pulmonary host defense against *Rhizopus* species

**DOI:** 10.1038/s41467-018-07301-y

**Published:** 2018-11-22

**Authors:** Angeliki M. Andrianaki, Irene Kyrmizi, Kalliopi Thanopoulou, Clara Baldin, Elias Drakos, Sameh S. M. Soliman, Amol C. Shetty, Carrie McCracken, Tonia Akoumianaki, Kostas Stylianou, Petros Ioannou, Charalampos Pontikoglou, Helen A. Papadaki, Maria Tzardi, Valerie Belle, Emilien Etienne, Anne Beauvais, George Samonis, Dimitrios P. Kontoyiannis, Evangelos Andreakos, Vincent M. Bruno, Ashraf S. Ibrahim, Georgios Chamilos

**Affiliations:** 1 Department of Medicine, University of Crete, Foundation for Research and Technology, 71300 Heraklion, Crete Greece; 2 Institute of Molecular Biology and Biotechnology, Foundation for Research and Technology, 71300 Heraklion, Crete Greece; 3 Laboratory of Immunobiology, Center for Clinical, Experimental Surgery, and Translational Research, Biomedical Research Foundation of the Academy of Athens, 11527 Athens, Greece; 4 Division of Infectious Diseases, Los Angeles Biomedical Research Institute, Harbor-University of California Los Angeles (UCLA) Medical Center, 1124 West Carson Street, St. John’s Cardiovascular Research Center, Torrance, CA 90502 USA; 5 Sharjah Institute for Medical Research, College of Pharmacy, University of Sharjah, PO Box 27272, Sharjah, UAE; 6 Institute for Genome Sciences, University of Maryland School of Medicine, Baltimore, MD 21201 USA; 7 CNRS, BIP (UMR 7281), IMM (FR 3479), Aix-Marseille Université, 31 chemin J. Aiguier, 13402 Marseille, France; 8 Unité des Aspergillus, Institut Pasteur, 75015 Paris, France; 9 Department of Infectious Diseases, Infection Control, and Employee Health, The University of Texas MD Anderson Cancer Center, Houston, TX 77030 USA; 10 David Geffen School of Medicine at UCLA, Los Angeles, CA 90095 USA

Correction to: *Nature Communications* 10.1038/s41467-018-05820-2; published online: 20 August 2018

The original version of this Article contained an error in the spelling of the author Emilien Etienne, which was incorrectly given as Emilien Ettiene. These errors have now been corrected in both the PDF and HTML versions of the Article.

