# Supplementary material for: Iron restriction inside macrophages regulates pulmonary host defense against Rhizopus species
Source: Nat Commun. 2018 Aug 20;9:3333. doi: 10.1038/s41467-018-05820-2 (PMC6102248; doi:10.1038/s41467-018-05820-2)
Supplement: Supplementary file 1 — Supplementary Information [file 41467_2018_5820_MOESM1_ESM.pdf]

Supplementary Figure 1

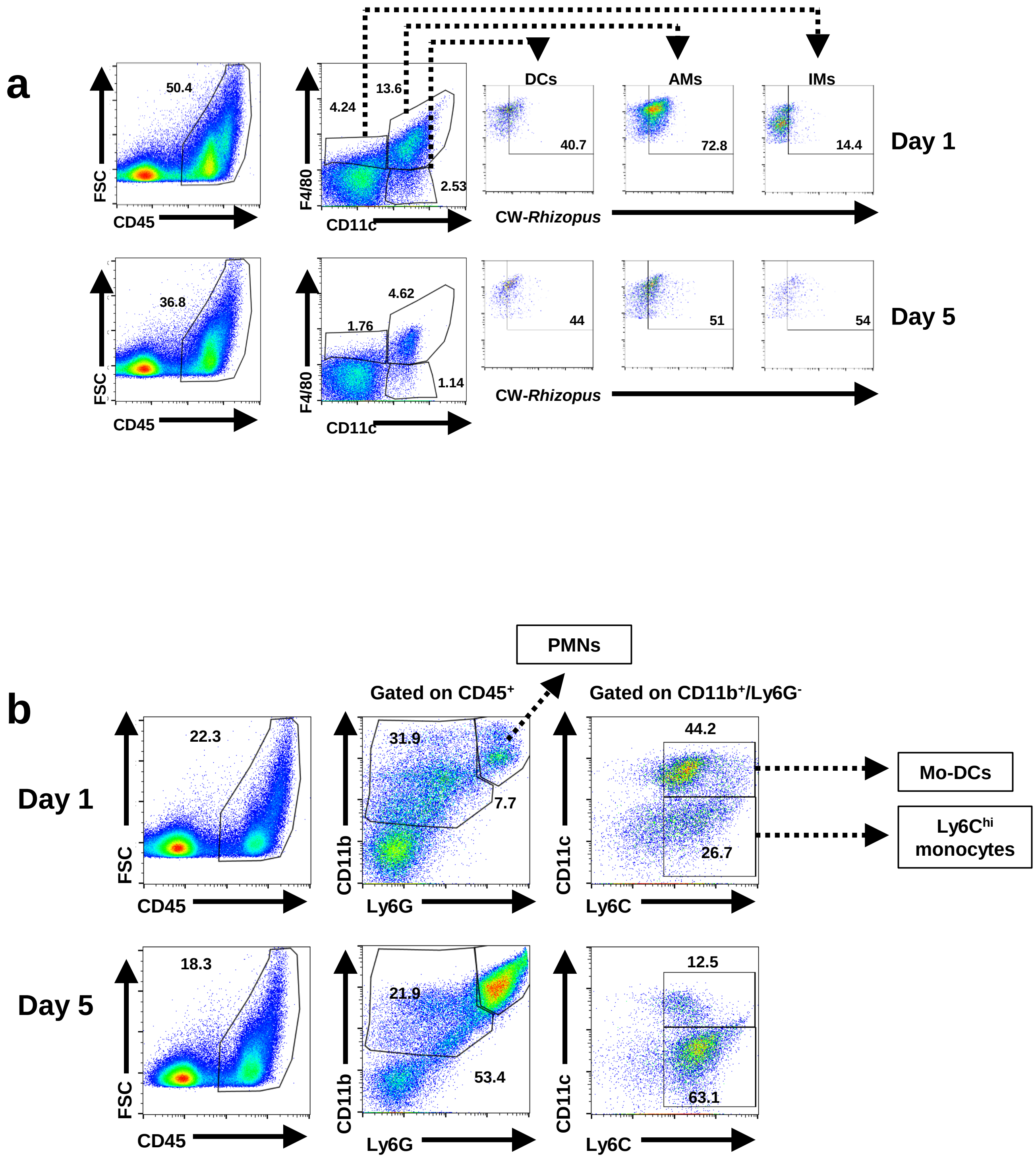

Supplementary Figure 1. FACS analysis of lung professional phagocytes following infection of C57BL/6 (B6) mice with *R. oryzae*. Gating strategy for identification of professional phagocytic cells in the lung following *R. oryzae* infection by FACS analysis. (**a**) Identification of AMs, IMs, and DCs based on CD11c and F4/80 expression of CD45+ cells. Association of fluorescent Brightener 28; CW-labeled R. oryzae conidia with each cell population is shown. (**b**) Identification of Neutrophils, monocyte-derived DCs (mo-DCs) and Ly6C^{hi} monocytes based on expression of CD11b, Ly6G, Ly6C, and CD11c of CD45+ cells.

Supplementary Figure 2

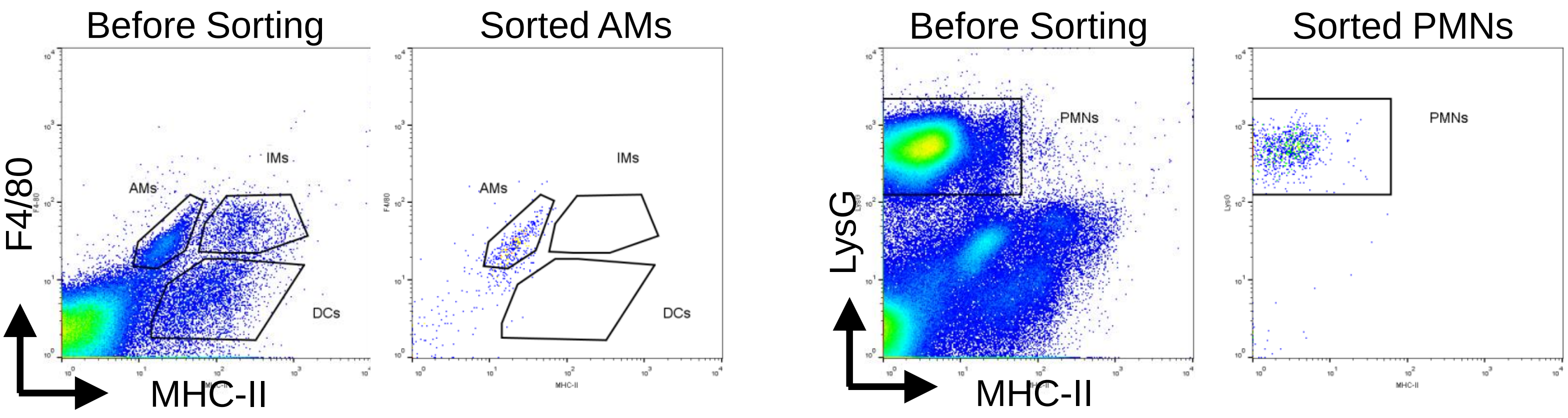

Supplementary Figure 2. Analysis of sorted AMs and Neutrophils (PMNs). AMs were sorted as CD45+/CD11c+/F4/80+/MHC-II lo cells. PMNs were sorted as CD45+/CD11b+/Ly6G+/MHC-II lo cells. Representative Data from one out of three independent experiments are shown.

Supplementary Figure 3

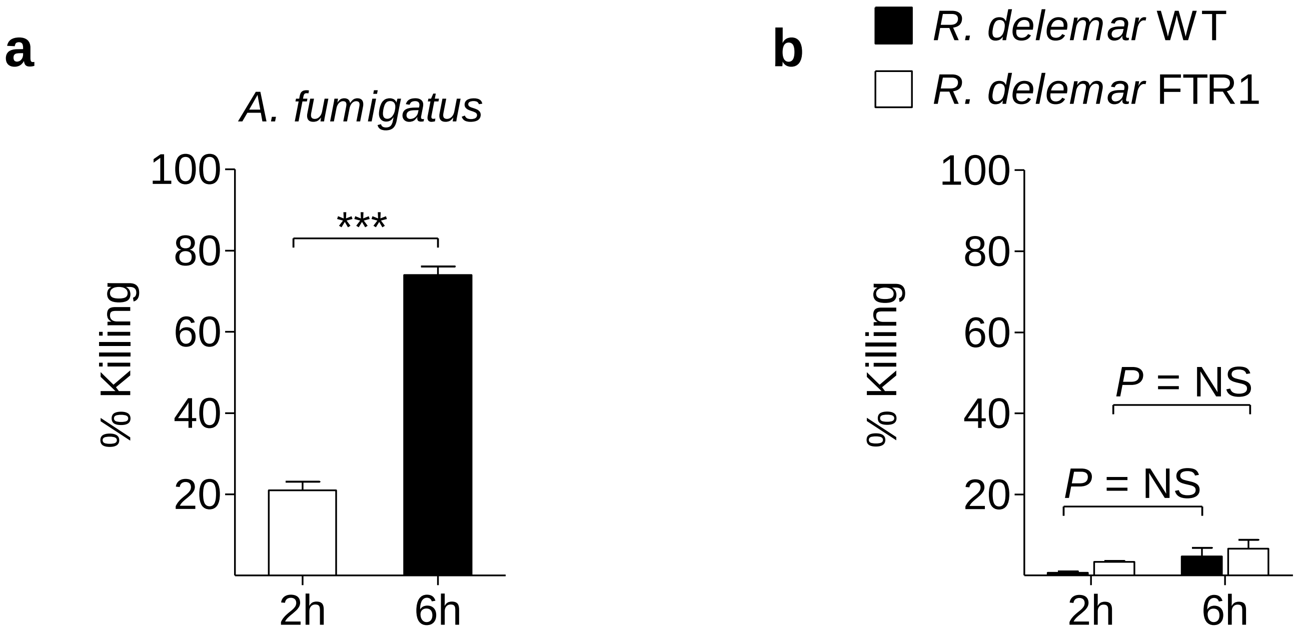

Supplementary Figure 3. *Rhizopus* spp are resistant to killing by BMDMs. BMDMs were infected with (**a**) *A. fumigatus* or (**b**) the indicated *Rhizopus* strains at a MOI of 1:1, unbound conidia were removed by extensive washing, cells were lysed at different time points of infection and the assessment of percentage of killing of conidia was counted based on germination rate of intracellular conidia. Germination of control conidia, inoculated in media in the absence of BMDMs, was always > 95%. Data on quantification of killing are presented as mean ± SEM of three independent experiments. *** *P*< 0.0001, Mann Whitney test

Supplementary Figure 4

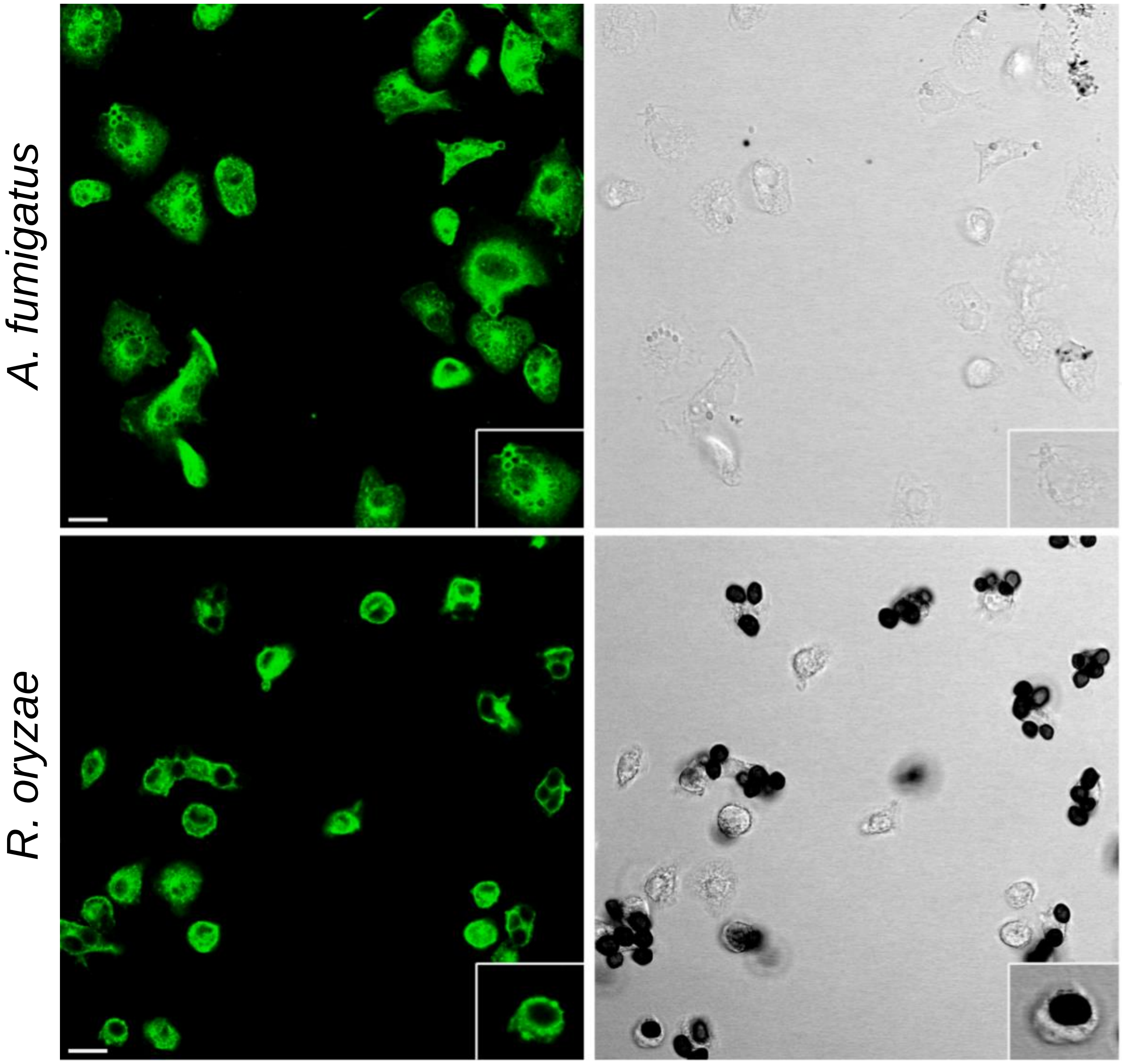

Supplementary Figure 4. Rab5 acquisition in *Rhizopus* containing phagosomes. BMDMs were infected with conidia of *A. fumigatus* or *Rhizopus* at a MOI of 3:1, fixed at 10 min of infection, stained for Rab5 and assessed by confocal imaging. Representative fluorescence images from one out of three experiments are shown. Rab5 recruitment is evident only in *A. fumigatus* phagosomes (inset).

Supplementary Figure 5

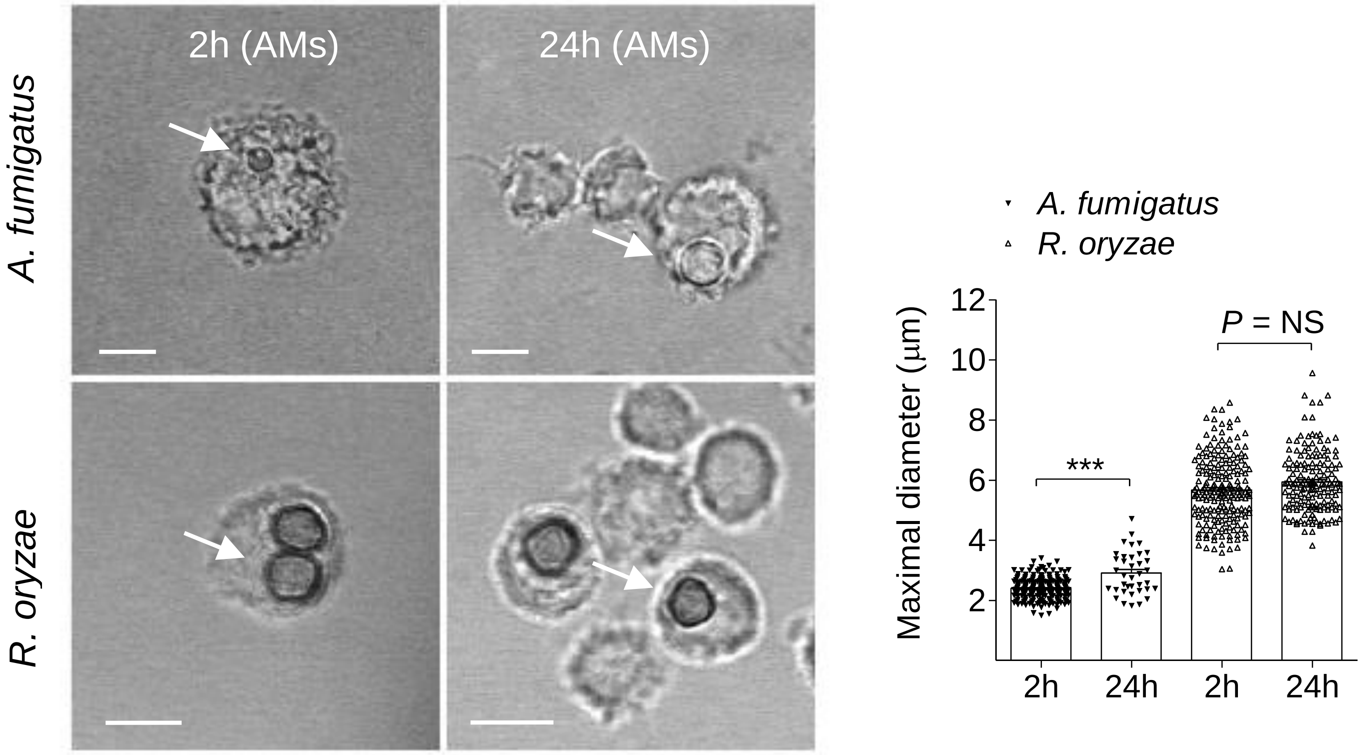

Supplementary Figure 5. ***In vivo*** **assessment of the size of conidia of** ***A. fumigatus*** **vs. Mucorales inside AMs.** GFP-LC3 mice were infected intratracheally with 5 X 10^6 conidia of *A. fumigatus* or *R. oryzae* and AMs were obtained by bronchoalveolar lavage at different time points (2 hr, 24 hrs) of infection. At the indicated time point cells were fixed, stained with anti-GFP and the conidial diameters of intracellular conidia was measured by confocal microscopy. Data on quantification of conidial diameter are presented from one out of three independent experiments. Each symbol represents the value of maximum diameter of individual fungal cell and horizontal bars represent the mean diameter. *** $P <$ 0.0001, Mann Whitney test. Representative DIC images from are shown.

Supplementary Figure 6

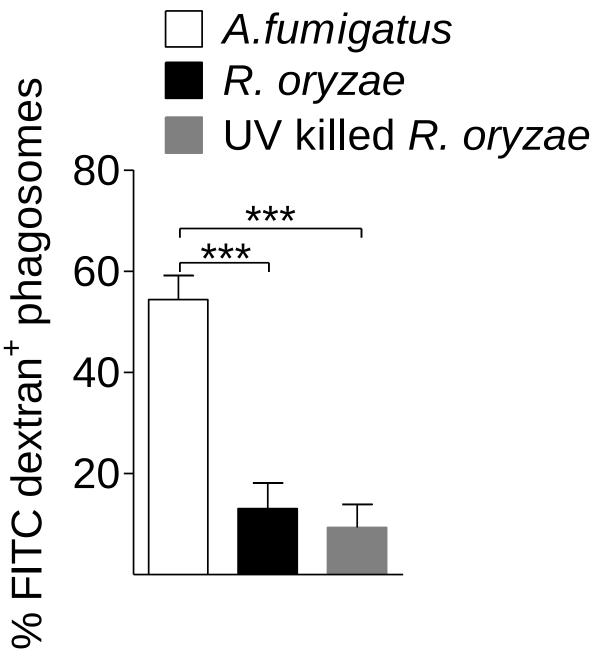

Supplementary Figure 6. UV killed conidia of *Rhizopus* retain the ability to block phagosome maturation. BMDMs preloaded overnight with FITC-dextran were infected with live conidia or UV killed conidia of *Rhizopus* or *A. fumigatus* conidia at a MOI of 3:1. Cells were fixed at 4h of infection and assessed by confocal imaging. Data on quantification of FITC-Dextran^{+} phagosomes are presented as mean ± SEM of three independent experiments. *** $P < 0.0001$, one-way ANOVA and Dunnett's multiple comparisons post-hoc test.

Supplementary Table 1

Experiment	Result
Solubility in water	Insoluble
Solubility in organic solvents (chloroform, methanol, ether, acetone, ethyl acetate and DMSO)	Insoluble
Color	Blackish brown
Solubility in 1 N (5%) KOH	Soluble
Precipitation in 0.1 -4N HCL	precipitated
Reaction for polyphenols with $FeCl_3$ test	Brown flocculent precipitate

Supplementary Table 1. Solubility of *R. delemar* pigment in different solvents.

Supplementary Figure 7

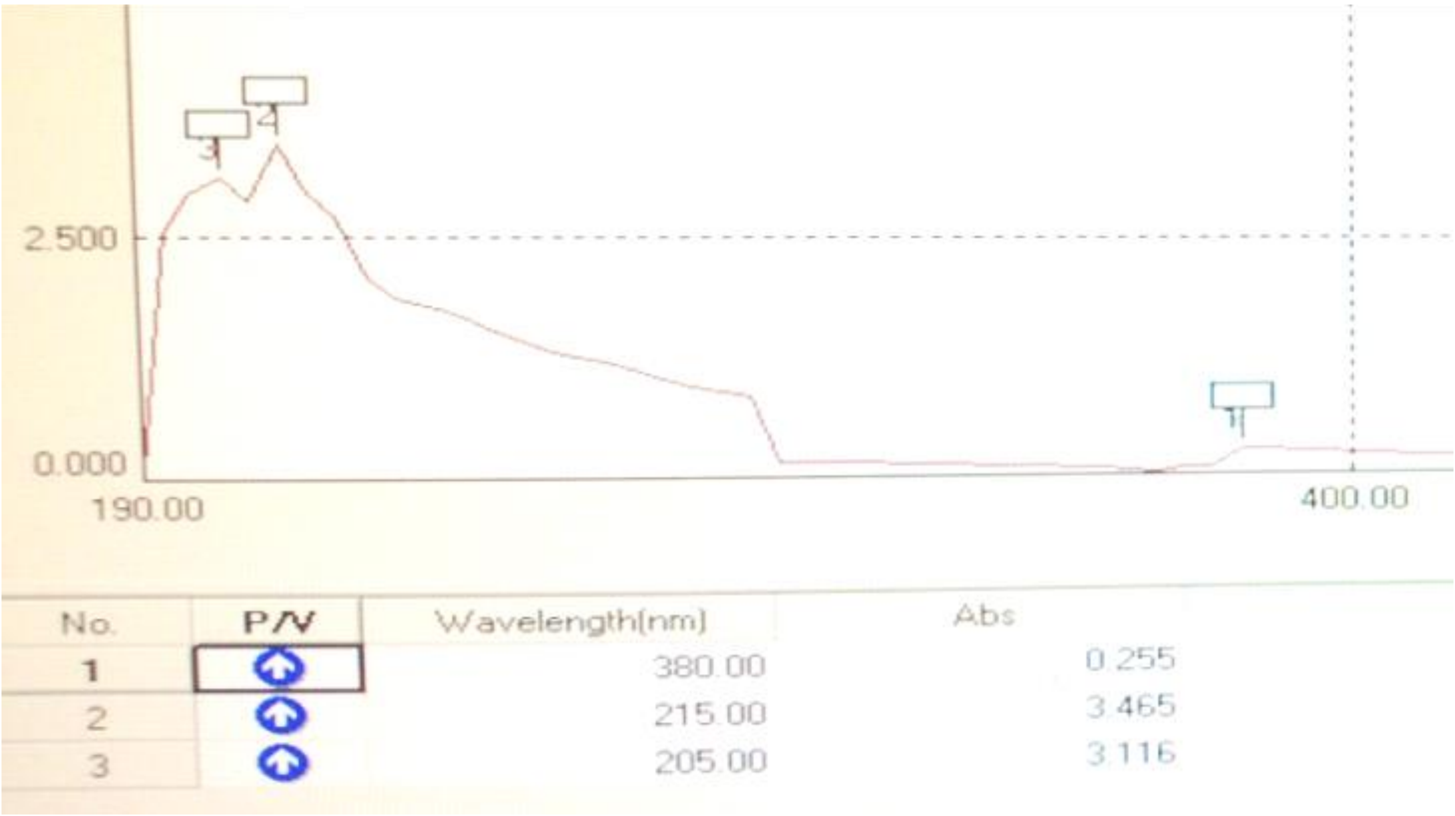

Supplementary Figure 7. UV absorbance of extracted pigment in 5% KOH. Two major peaks at 215 and 205 nm indicative of melanin pigment-like substance.

Supplementary Figure 8

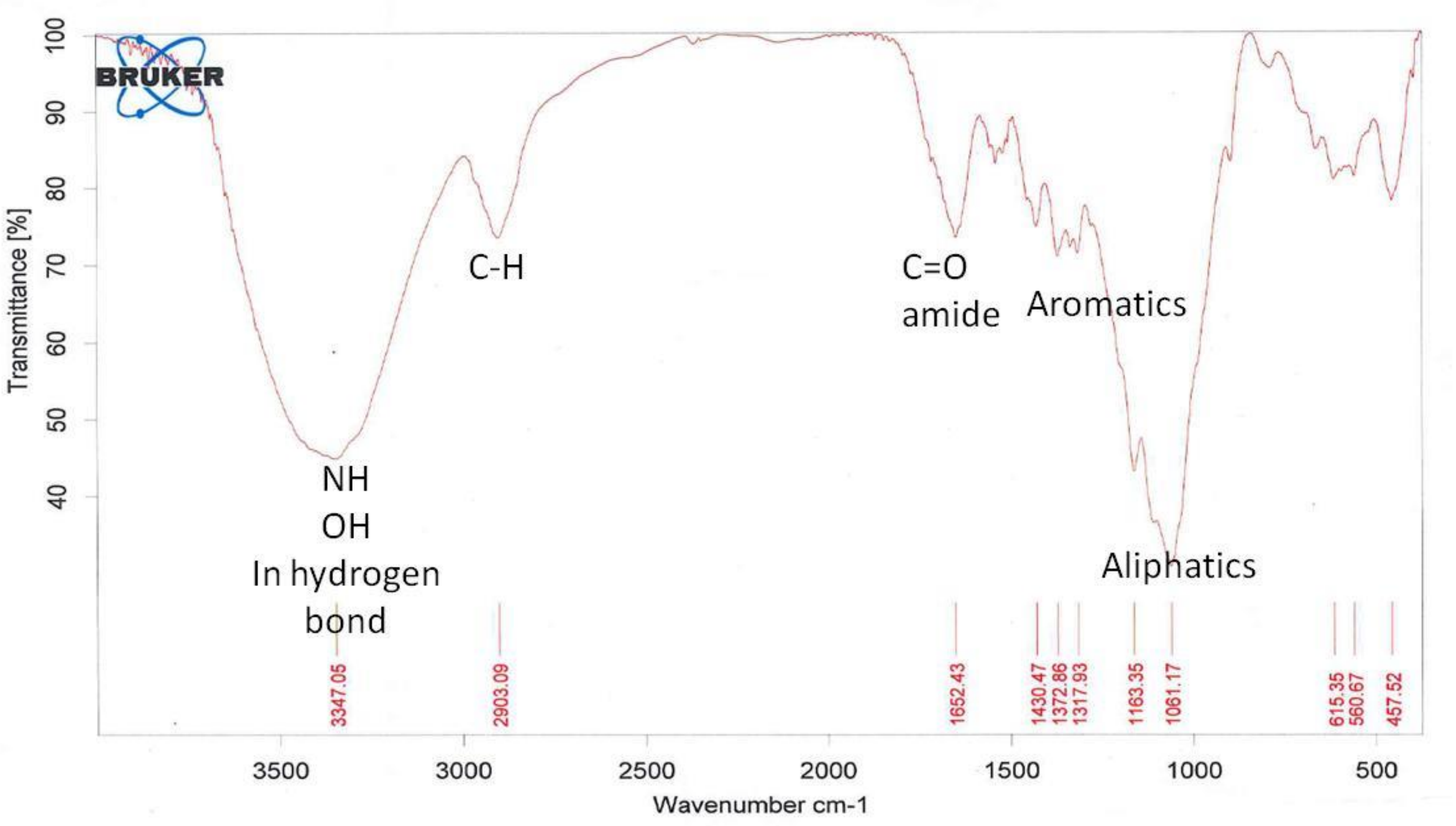

Supplementary Figure 8. IR spectroscopy of the pigment in KBr disc. The signals in the 3600-2800 cm^{-1} area are attributed to the stretching vibrations of (O-H and N-H) of the carboxylic acid. Phenolic and aromatic amino functions presents in the indolic and pyrrolic systems. 1750 and 1550 cm-1 the bending vibrations of the C=O double bond (COOH) can be found as well as the ones of the carbon-carbon double bond, the carbon-nitrogen bond of the aromatic system and of the carbon-oxygen double bond of those carboxylic functions that are interested in the bond formation with the metal ions. The OH bending of the phenolic and carboxylic groups were present in the 1400-1300 cm^{-1} area. The out-of-plane bending of the aromatic carbon hydrogen bond can be found in the 700-600 cm^{-1} area.

Supplementary Figure 9

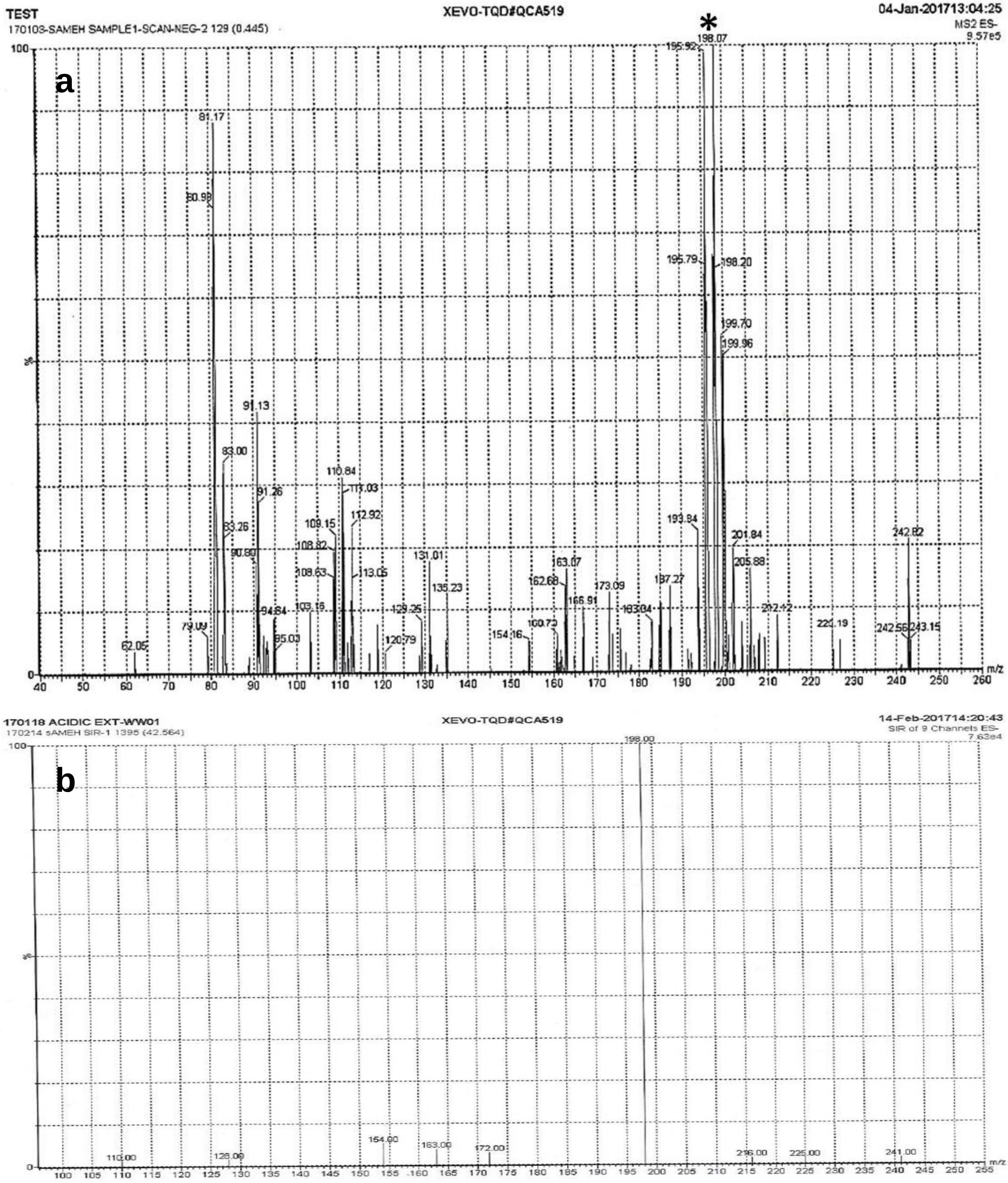

Supplementary Figure 9. LC-MS analysis of the alkaline hydrolyses product. **(a)** LC-MS scan for major peaks of the pigment hydrolyses products. (**b)** Mass fragmentation pattern of the M/Z 198 peak.

Supplementary Figure 10

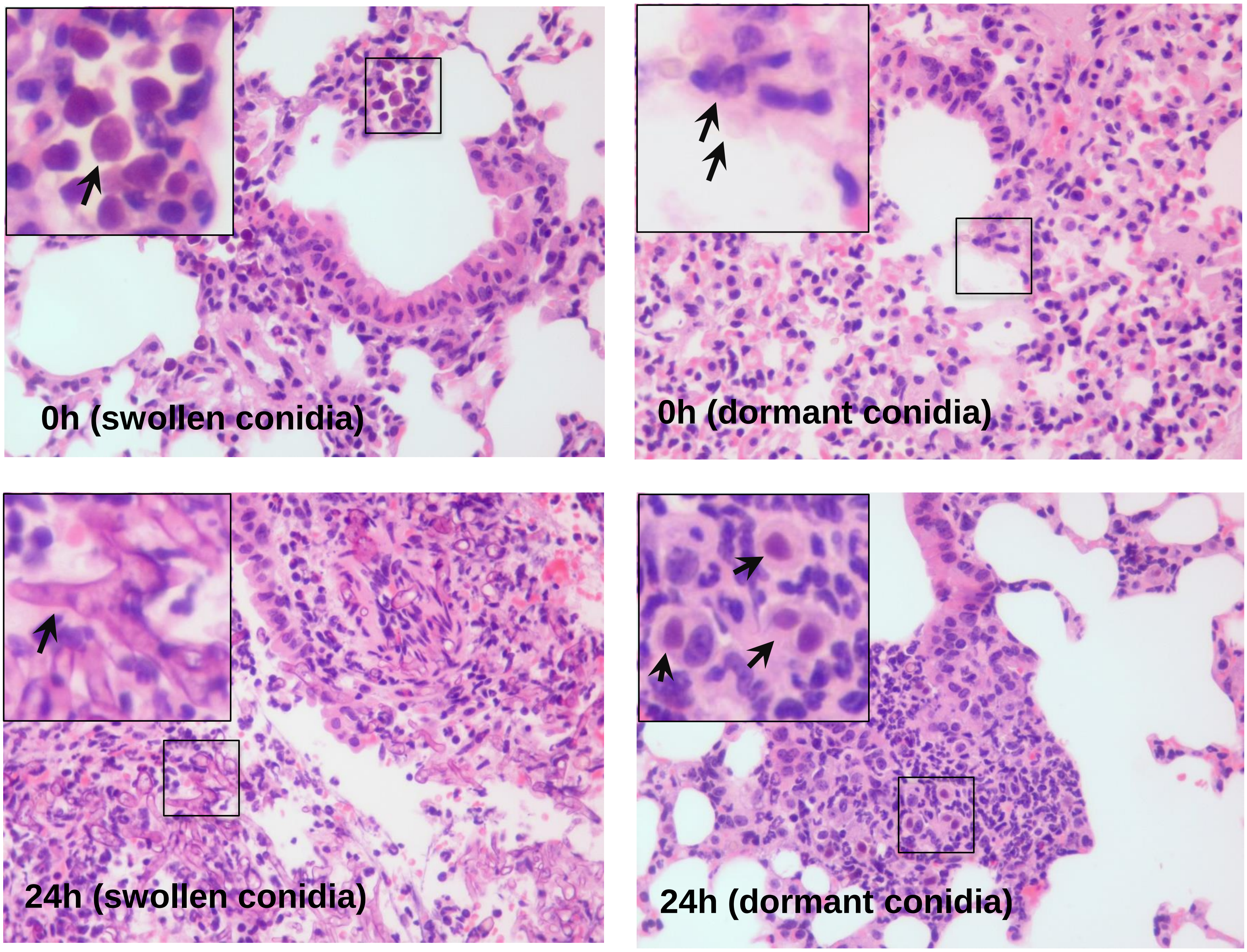

Supplementary Figure 10. Histopathology of lungs from mice infected with dormant or swollen *Rhizopus* conidia. Representative photomicrographs of the lungs from C57BL/6 (B6) mice infected intratracheally with 5 x 10^6 dormant or swollen conidia (incubated in media for 4h) of *R. oryzae* at 0h at 24 h of infection. Lungs were removed, fixed and stained by H&E. The size of swollen as compared to dormant conidia at the time of infection (0h) is shown in insets. At 24h of infection there is evidence of extensive *R. oryzae* hyphal growth in the lungs of the mice infected only with swollen conidia. Notably, there is no evidence of germination of dormant conidia, which predominately reside inside lung macrophages. original magnification X400. Arrows indicate fungal cells

Supplementary Figure 11

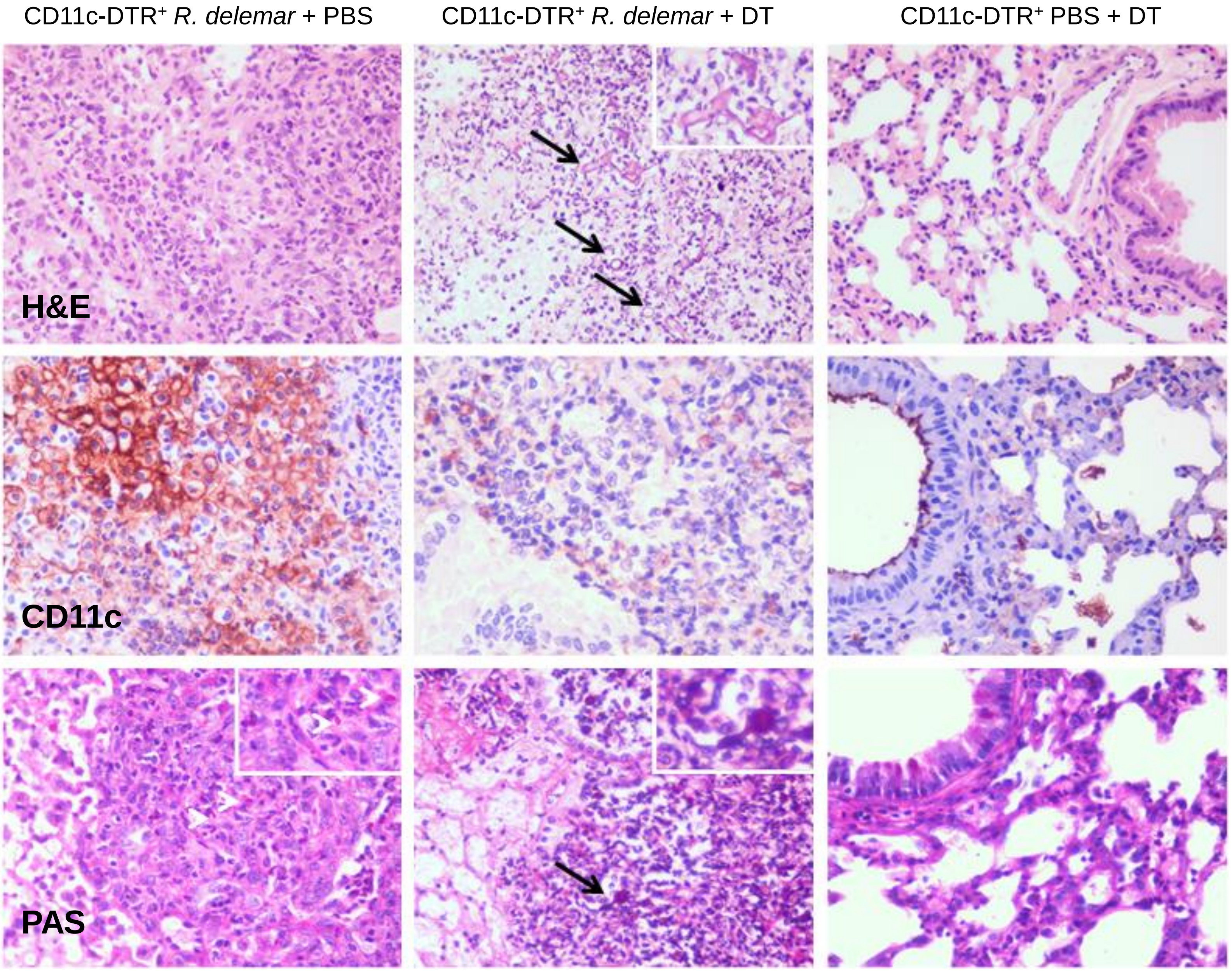

.

Supplementary Figure 11. Histopathology of lungs from CD11c-DTR mice. Representative photomicrographs of the lungs from CD11c-DTR$^+$ mice intranasally inoculated with 40 µL PBS or 20 ng/g bwt of diphtheria toxin (DT) in 40 µL PBS and 48 h after infected with 5 x 10^6 swollen conidia of *R. oryzae* or treated with PBS at 24 h of infection. Lungs were stained by H&E or PAS or IHC for CD11c. There is evidence of extensive *R. oryzae* hyphal growth with angioinvasion, intense neutrophil infiltration and necrosis, consistent with fungal pneumonia in the lungs of mice following CD11c+ cell depletion. White arrowheads: spores; Black arrows: hyphae; H&E: eosin and hematoxylin stain; PAS: Periodic- acid Shift stain; hematoxylin as counterstain; original magnifications X200, X400
